# A Randomized Controlled Multicenter Trial of an Investigational Liquid Nutritional Formula in Women with Cyclic Breast Pain Associated with Fibrocystic Breast Changes

**DOI:** 10.1089/jwh.2017.6406

**Published:** 2018-03-01

**Authors:** Robert E. Mansel, Tapas Das, Geraldine E. Baggs, Michael J. Noss, William P. Jennings, Jay Cohen, David Portman, Mario Cohen, Anne Coble Voss

**Affiliations:** ^1^ Department of Surgery, Cardiff University, Cardiff, Wales, United Kingdom.; ^2^ Research and Development, Abbott Nutrition, Columbus, Ohio.; ^3^ Synexus (formerly Radiant Research), Cincinnati, Ohio.; ^4^ Synexus (formerly Radiant Research), San Antonio, Texas.; ^5^ Envision Physician Services, Discovery Clinical Research, Plantation, Florida.; ^6^ Sermonix Pharmaceuticals, LLC, Columbus, Ohio.; ^7^ Greater Hartford Women's Health Association, West Hartford, Connecticut.

**Keywords:** fibrocystic breast changes, gamma-linolenic acid, iodine, mastalgia, selenium

## Abstract

***Objective:*** A randomized, multicenter, controlled double-blind trial was performed in women with cyclic breast pain (mastalgia) associated with fibrocystic breast changes (FBCs) to determine whether a nutritional formula reduced breast pain and/or nodularity.

***Study Design:*** Women were randomized to receive a specifically designed liquid formulation (*n* = 93) (1 g gamma-linolenic acid [GLA], 750 μg iodine, and 70 μg selenium) or control formula (*n* = 95) (without GLA, iodine, and selenium) daily for three cycles. Women recorded breast pain, medications, and menstrual signs daily using interactive voice-response system. Nodularity was determined by physical breast examination.

***Results:*** Breast pain scores decreased similarly in the experimental (−32.2%) and control (−33.1%) groups (*p* = 0.64). Nodularity was reduced in the experimental, but not the control group (*p* = 0.03). Among women who continued pain medication, the amount was reduced in the experimental group relative to controls (*p* = 0.02).

***Conclusion:*** Women with FBC using the formula containing GLA, iodine, and selenium experienced reduced nodularity and in those women who took over-the-counter breast pain medication, a decrease in the quantity of pain medication was observed.

## Introduction

Fibrocystic breast change (FBC) is a benign disorder of breast physiology that can result in lumpiness or nodule formation. The prevalence of breast nodularity in a random population-based sample of women in the community and hospital is 62%.^1^ In FBC, breast nodularity is due to small breast masses (fibrosis) and breast cysts. Nodularity does not increase breast cancer risk, but it can make a mammogram more difficult to read or interpret. On clinical examination, cysts are palpable as discrete, rounded lumps, which cannot be reliably distinguished from solid tumors. Fibrosis is composed of fibrous and epithelial tissue and present as firm, nontender, palpable lumps. Nodularity is often associated with cyclic menstrual related breast pain, or cyclic mastalgia, giving rise to patient fear of breast cancer and discomfort severe enough to affect quality of life.^2^

Current treatment options for FBC are limited and not fully effective. Over-the-counter (OTC) pain medication (acetaminophen, nonsteroidal anti-inflammatory drugs [NSAIDS]) may be treatment options for breast pain. Prescription hormonal treatment medications—tamoxifen (selective estrogen receptor modulator with estrogen agonist–antagonist properties),^3^ danazol (attenuated androgen),^4^ and goserelin (luteinizing hormone-releasing hormone analog)^5^—are expensive, have short- and long-term adverse side effects, and can only be used for 2- to 6-month periods. Results of studies suggested benefits from nutritional interventions for treatment of cyclic mastalgia; dietary intake of gamma-linolenic acid (GLA), usually as evening primrose oil,^6^ or various forms of iodine were reported to reduce breast pain.^7^ However, pain relief by iodine was only achieved by provision of iodine in a nondietary form and in amounts that exceed the recommended upper limit for safe dietary intake.^8^

In this study, the hypothesis tested was that GLA and iodine may complement each other in providing relief from nodularity and mastalgia by modulating pathways associated with estrogen and/or thyroid hormone metabolism.^9^ Selenium was added to help minimize potential adverse effects of increased iodine intake on the thyroid.^10^ Support for the proposed combination of these ingredients comes from *in vitro* studies that show a synergistic effect of GLA, iodine, and selenium. Modulation of the function of human endothelial cell intercellular tight junctions (TJs),^11^ and the complete reversal of the effects of estradiol on paracellular permeability and the function of TJ in human breast cancer cells were observed with the unique combination of GLA, iodine, and selenium.^12^ The underlying mechanism of strengthening the TJ function by these ingredients is to relocate properly the important TJ proteins (claudin-5, occludin, and ZO-1) to the cell–cell junction, thus preventing tissue swelling and edema, the etiology of breast nodularity.

The purpose of this study was to evaluate a nutritional liquid formulation containing GLA (1 g from borage oil), iodine (750 μg in the form of potassium iodide), and selenium (70 μg in the form of sodium selenate) compared to an isocaloric, isonitrogenous liquid formula without GLA, iodine, or selenium on nodularity and cyclic breast pain in women with FBC. Both control and experimental nutritional liquid formulations were made specifically for this trial.

## Materials and Methods

### Study design

**FIG. 1.**
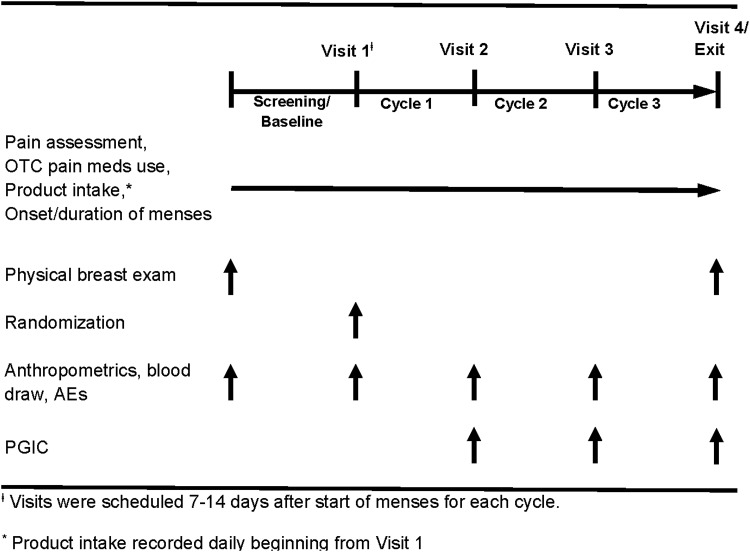
Study design.

The study was a multicenter, prospective, randomized, double-blind, controlled parallel-group study (Fig. 1). Women were instructed to consume 4 fluid ounces (fl oz) liquid nutritional formula daily of the allocated intervention beginning with visit 1. Patient assessments were performed 7 to 14 days after the start of menstrual cycles 1 and 2 and 10 to 20 days after the start of cycle 3 or at study discontinuation, and included intake of allocated intervention. Self-reported breast pain was documented using an 11-point Likert scale; peak pain sum scores were obtained by adding the five highest pain scores during the most painful, consecutive 10-day period of the menstrual cycle. Use of supplemental medications(s) for control of breast pain, type of medication and number of pills, and onset and duration of menses were recorded daily using an interactive voice response system (IVRS) (ClinPhone, Inc., Nottingham, United Kingdom). Breast nodularity was determined by physical breast examination performed by a healthcare professional at screening and baseline and was scored using a validated 4-point scale (none/no nodules, few/one nodule, several nodules, or numerous nodules) that supported the evaluation of danazol.^1^ Other clinical assessments included determination of weight, medication/dietary supplement use, and adverse events. Women evaluated their gastrointestinal tolerance and Patient Global Impression of Change (PGIC) at cycles 1, 2, and 3. Blood was drawn at baseline and cycles 1, 2, and 3 and analyzed by Quest Diagnostics (Collegeville, PA). The protocol received approval from the appropriate site Institutional Review Board. All participants provided written informed consent before any participation in the study.

### Objectives

The primary objective of the study was to evaluate the impact of a once-daily nutritional formula on cyclic breast pain in women with FBC. Using an 11-point Likert scale, peak pain sum scores were obtained by adding the five highest pain scores during the most painful, consecutive 10-day period of the menstrual cycle.

The secondary objectives of the study were physician assessment of breast nodularity by physical breast examination^13^ at baseline and at the end of the study; the amount, type, and frequency of nonprescription medication(s) used throughout the study period for control of breast pain; change in weight; and PGIC in response to treatment. Measures of safety and tolerance were determined for both the control and experimental nutritional formulations.

### Participants

Eligible women were between the ages of 18 and 55 years with a history of cyclic mastalgia associated with FBC. Women included in the study were generally healthy, not pregnant, had regular menstrual cycles of 21–35 days, and had a body mass index between 18.5 and 34 kg/m^2^. Sexually active women had to agree to maintain the same, effective form of birth control throughout the study. Women using hormonal contraceptive medications had to have taken the same (or similar) medication and dose for three cycles before enrollment. Women were excluded if they were pregnant; had a history of bilateral oophorectomy or hysterectomy; used hormone replacement therapy; had a suspicious or abnormal mammogram; chronically used analgesic, diuretic, anti-inflammatory, or iodine-containing medications (thyroid hormone replacement less than 125 μg per day was allowed); had history of reduction mammoplasty, breast, or thyroid cancer; had active thyroiditis; used iodine or supplements containing GLA (evening primrose or borage oil), or took high-dose vitamin or nutritional supplements (routine vitamin/mineral supplements were allowed).

### Study treatments

Women were randomized (1:1) to receive daily a 4 fl oz ready-to-drink liquid nutritional formula containing 100 kcal, 1 g GLA, 750 μg iodine, and 70 μg selenium (experimental formula) (Abbott Nutrition, Columbus, OH), or an isonitrogenous, isocaloric formula prepared with high-oleic safflower oil (HOSO) and without GLA, iodine, or selenium (control formula) (Abbott Nutrition). The nutritional composition of the experimental and control formulations is shown in Table 1.

**Table 1.** Control and Experimental Nutritional Formula Composition

*Nutrient (per 4 fluid ounces)*	*Control*	*Experimental*
Energy, kcal	100	100
Protein, g	5	5
Carbohydrate, g	9	9
HOSO, g (fat)	5	0
Borage oil,^a^ g (fat)	0	5
Vitamin D, IU	100	100
Vitamin K, μg	20	20
Folic acid, μg	400	400
Vitamin B_6_, mg	2	2
Vitamin B_12_, μg	6	6
Sodium, mg	154	154
Potassium, mg	92	92
Chloride, mg	154	154
Calcium, mg	250	250
Phosphorus, mg	250	250
Magnesium, mg	100	100
Iodine, μg	0	750
Selenium, μg	0	70

^a^ 5 g borage oil provided 1 g GLA.

GLA, gamma-linolenic acid; HOSO, high oleic safflower oil.

### Blinding

The women, the investigators, and their staffs were blinded to the identity of the study products during the trial. In the development of experimental and control products, every attempt was made to minimize differences in taste, texture, and appearance. The same four flavors were available in experimental and control products and all packaging was identical in appearance, identified only by product codes that were blinded to study subjects, investigators, and sites.

### Randomization

Following the baseline period of pain assessment, subjects with a primary outcome peak pain sum score of at least 15 out of a possible 50 using the Likert pain scale on each day, were randomized if they were at least 70% compliant in reporting their daily pain during the screening period. A central randomization approach was used to assign subjects to treatment groups in a 1:1 ratio within each study site. As the primary outcome potentially could be influenced by hormonal contraceptive use, balance on treatment groups was assured through a dynamic allocation procedure. Allocation concealment was achieved through utilization of a validated centralized randomization system (ClinPhone, Inc., Nottingham, United Kingdom).

### Sample size

To detect a 2-point treatment group difference in change from baseline using an 11-point Likert pain scale, a total of 222 patients would be needed with an alpha of 0.01, two-sided test, and with 95% power. A common estimate of standard deviation of 3.5 is assumed. Assuming a 20% attrition rate, a total of 278 subjects were required. The sample size calculation was done using nQuery Advisor 4.0 software.

### Statistical methods

The change from screening to cycle 3 in the average peak pain sum scores was analyzed using analysis of variance with factors for treatment, site, and screening (baseline) average peak pain sum scores as a covariate. Pain medication data were analyzed using Wilcoxon rank sum test.

Change in nodularity rating from baseline to end of the study was assessed using Bowker's test of Symmetry for rxr tables. The Spearman's rank-order correlation coefficient between nodularity and average peak pain sum score at end of the study was computed *post hoc*.

Repeated measures analysis was used to analyze data on weight change with factors for treatment, site, cycle, treatment by cycle interaction, and screening (baseline) weight as a covariate.

SAS^®^ version 9.2 and 9.4 was used in all analyses. Statistical significance was declared when the *p*-value ≤0.05. Continuous data are expressed as mean ± standard error.

An interim efficacy analysis was conducted using the group sequential approach of Lan and DeMets,^14^ with a spending function for the type I error corresponding to O'Brien-Fleming.^15^ The interim result indicated there was a low probability of demonstrating a statistically significant difference between control and treatment groups in the primary endpoint at the end of the trial. Hence, a decision was made to stop the trial, but before the results on concomitant nonprescription pain medication use and breast nodularity were known (see Results section below). When the trial was terminated, approximately 67% of the patients (including overruns) had been randomized into the study. Subsequent analyses of secondary and supportive data were made and results reported here.

## Results

**FIG. 2.**
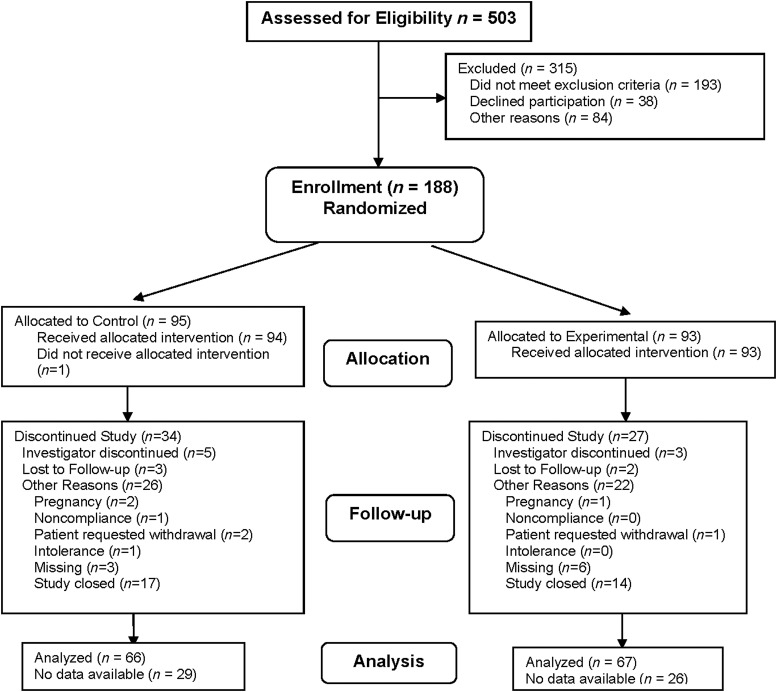
Participant flow diagram.

Subjects (control *n* = 95 and experimental *n* = 93) were recruited from 21 centers distributed across the United States from July 20, 2004, to November 2, 2005 (participant flow diagram Fig. 2). The groups were well matched on baseline characteristics (Table 2) with no significant differences between groups. Compliance with the nutritional intervention was 90.4% in the experimental group and 82.7% in the control group. Subjects were considered compliant with treatment if they consumed ≥70% of the required amount of study product (reported daily by IVRS).

**Table 2.** Demographic and Baseline Data

*Variable*	*Control (*n* = 95) Mean ± SEM (*n*)*	*Experimental (*n* = 93) Mean ± SEM (*n*)*
Age (years)	36.5 ± 1.0 (94)	36.3 ± 0.9 (90)
Weight (pounds)	144 ± 2.6 (91)	145.2 ± 2.9 (91)
Age at menarche (years)	12.8 ± 0.2 (93)	12.4 ± 0.2 (91)
Gravidity	1.8 ± 0.2 (94)	1.9 ± 0.2 (91)
Parity	1.1 ± 0.1 (92)	1.3 ± 0.1 (90)
Average months of breastfeeding	3.3 ± 0.7 (94)	3.4 ± 0.7 (90)
Duration of FBC symptoms (years)	8.5 ± 0.8 (94)	9.5 ± 0.8 (89)
Average peak pain during screening	5.3 ± 0.2 (93)	5.2 ± 0.2 (91)

	*Percent (*n*)*	*Percent (*n*)*
Nonprescription pain medication	58.9 (56/95)	51.6 (48/93)
Smoker	21.3 (20/94)	20.9 (19/91)
Contraception	77.7 (73/94)	83.5 (76/91)
Oral contraception	11.7 (11/93)	18.7 (17/91)
Consumes soy products	36.6 (34/93)	43.3 (39/90)
Consumes caffeine	90.3 (84/93)	95.6 (86/90)
Previous OB/Gyn surgery	58.5 (55/94)	57.1 (52/91)

FBC, fibrocystic breast change; OB/Gyn, obstetrical/gynecological; SEM, standard error of the mean.

### Outcomes

The primary outcome measure, average peak breast pain scores, decreased from baseline similarly in both groups (5.16 ± 0.18 at the baseline and 3.50 ± 0.24 at the end; 32.2% experimental, 5.34 ± 0.17 at the baseline and 3.57 ± 0.25 at the end; 33.1% control; *p* = 0.64). Women on oral contraceptives exhibited nonsignificant larger reductions in pain scores compared with those using other contraceptives or not sexually active (Table 3). After 3 cycles, women consuming the experimental formula showed improved breast nodularity from baseline (*p* = 0.03), while women consuming the control formula did not (Table 4). There was a 53% relative decrease in the number of women with severe nodularity (numerous nodules) and an 83% increase in those with no breast nodules in the experimental group (Table 4). In contrast, in the control group, the number of women with severe nodularity (numerous nodules) decreased by 19% and women without breast nodules decreased by 30%.

**Table 3.** Mean Breast Pain Scores by Oral Contraceptive Use

	*Mean breast pain*			*Change in mean breast pain from screening*		
*Cycle/contraceptive use*	*OC user*	*Other contraceptive user*	*Not sexually active*	*OC user*	*Other contraceptive user*	*Not sexually active*
0	5.51 ± 0.31 (28)	5.19 ± 0.15 (123)	5.27 ± 0.28 (35)			
1	4.69 ± 0.33 (28)	4.58 ± 0.17 (121)	4.84 ± 0.31 (34)	−0.81 ± 0.27 (28)	−0.64 ± 0.16 (121)	−0.44 ± 0.27 (34)
2	3.91 ± 0.35 (26)	3.81 ± 0.19 (100)	3.83 ± 0.33 (30)	−1.52 ± 0.29 (26)	−1.29 ± 0.21 (96)	−1.28 ± 0.37 (30)
3	3.17 ± 0.50 (18)	3.54 ± 0.21 (87)	3.77 ± 0.38 (28)	−2.20 ± 0.56 (18)	−1.55 ± 0.23 (87)	−1.37 ± 0.41 (28)

Mean ± SEM (*n*).

OC, oral contraceptive.

**Table 4.** Change in Breast Nodularity After Three Cycles

	*Control* p*-value*^a^* = 0.64*			*Experimental* p*-value*^a^* = 0.03*		
	*None*	*Mild to moderate*	*Severe*	*None*	*Mild to moderate*	*Severe*
Baseline, *N* (%)	10 (15)	38 (59)	16 (25)	6 (9)	44 (66)	17 (25)
End of study, N (%)	7 (11)	44 (69)	13 (20)	11 (16)	48 (72)	8 (12)
% Change in *N*	−30%	+16%	−19%	+83%	+9%	−53%

^a^ *p*-Value based on Bowker's test of Symmetry for rxr tables.

At the end of the third cycle, there was a significant correlation between breast pain and nodularity (Spearman's correlation *r* = 0.218; *p* = 0.018) among all subjects who had pain and nodularity assessed (*n* = 118), indicating that more nodules were associated with greater breast pain (Table 5).

**Table 5.** Average Peak Pain Score Versus Breast Nodularity at the End of Three Cycles

*Nodularity*	*Average peak pain ± standard error*
None (*n* = 17)	2.85 ± 0.53
Few (*n* = 43)	3.40 ± 0.28
Several (*n* = 37)	3.59 ± 0.34
Numerous (*n* = 21)	4.40 ± 0.48

**FIG. 3.**
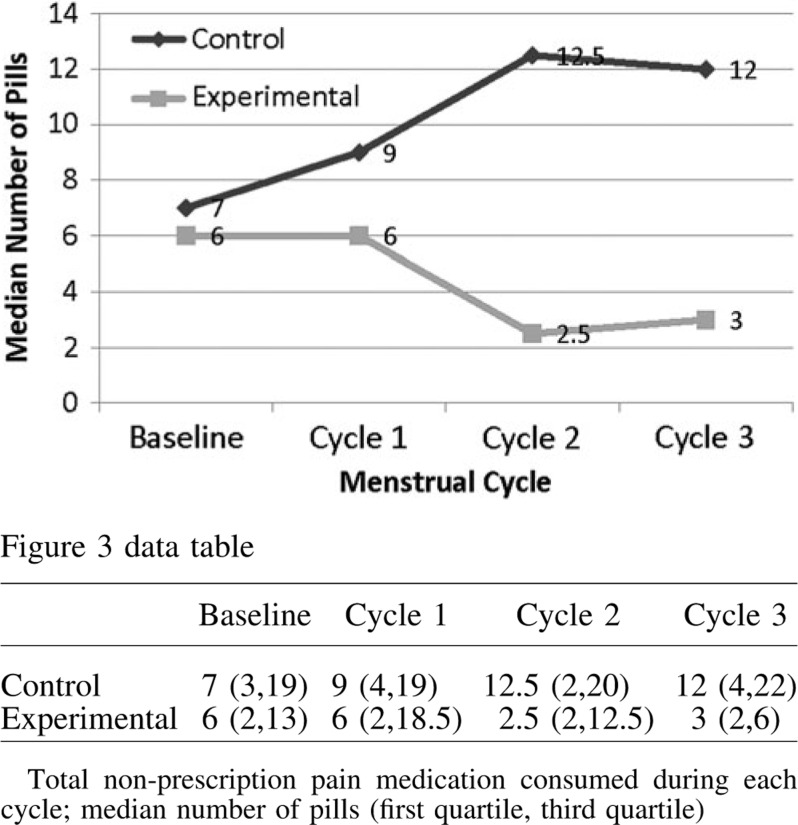
Total nonprescription pain medication consumed during each cycle for breast pain.

The percentage of women taking nonprescription analgesics and/or nonsteroidal anti-inflammatory medications for breast pain at the end of the study was similar in both groups [*n* = 19 (23.7%) experimental, *n* = 18 (23.2%) control; *p* = 0.94]. However, among women who continued to take any type of OTC pain medication for breast pain during the last menstrual cycle, the median (*p* = 0.014; Fig. 3) or average (*p* = 0.02) number of pills decreased in the experimental group. Compared with baseline, women in the experimental group, who chose to use pain medications, had a 50% decrease in the median amount of pills by the end of cycle 3. The women in the experimental group using only ibuprofen to control their breast pain decreased the amount of pain medication by the second menstrual cycle (*p* = 0.03; data not shown).

In consideration of the potential impact on the reported severity of breast pain as a consequence of the difference in the amount of OTC pain medications used by women in the control and experimental groups, the adjusted breast pain score (APS), defined *post hoc*, captures the amount of pain managed per unit of pain medication taken. This APS indicated a higher mean response rate (*i.e.*, similar pain managed with less pain medication) in the experimental group than the control group by the third cycle (*p* = 0.03; Table 6).

**Table 6.** Adjusted Breast Pain Scores

	*Change of adjusted breast pain from screening*^a^		
*Cycle*	*Control*	*Experimental*	p*-Value Wilcoxon rank sum test*
1	−0.46 ± 0.19 [−0.18]	−0.54 ± 0.31 [−0.32]	0.65
2	−0.20 ± 0.23 [−0.17]	−0.32 ± 0.33 [−0.08]	0.86
3	−0.46 ± 0.18 [−0.66]	0.25 ± 0.22 [0.0]	0.03

Adjusted Breast Pain Score is average peak pain score adjusted for nonprescription pain medication use; mean ± SEM (*n*); median values are in brackets.

^a^ Change from baseline by dividing the breast pain scores by the average number of pills taken for pain during the 5 worst days of pain for that cycle for each subject.

Both groups reported similar average weight at baseline (control 144 pound [lb] and experimental 145.2 lb; Table 2). Weight changes during the trial were not significantly different between groups (0.57 ± 0.38 lb, experimental; 0.12 ± 0.39 lb, control; *p* = 0.38). PGIC scores at the end of the study were similar between groups with 71% of women in the control group and 76% of women in the experimental group rating their condition as “improved.” Approximately half the women in each group described their condition as “much or very much improved.”

### Adverse events

The nutritional formulations used in the study were well tolerated and the number of subjects reporting one or more significant, but not serious, adverse events was similar in the control and experimental groups (*p* = 0.46). There were no differences between the groups for specific measures of gastrointestinal tolerance. There was an increase in thyroid-stimulating hormone (TSH) levels in five women in the experimental group, but none in the control group. Out of five women, four reported adverse events (AEs) with mild severity and possibly attributable to the study formula. None of these women were clinically symptomatic, their thyroxine (T4) levels were all within the normal range, and all continued with the study without interruption of treatment. TSH levels resolved spontaneously, returning to normal in three of the four women within 1–2 menstrual cycles.

## Discussion

The randomized controlled study evaluated the effect of a novel nutritional formula containing a combination of GLA, iodine, and selenium to reduce cyclic breast pain and nodularity in women with FBC. Findings from this study provide additional knowledge and experience of nonpharmacological, nutritional interventions for women with FBC and associated cyclic mastalgia. In this study, the primary endpoint was not achieved, no differences were observed between the control and experimental groups with regard to reduction in cyclic breast pain, a finding possibly attributable to a placebo effect that is commonly reported in studies of cyclic mastalgia.^16^ In an analysis of American trials of pain medications, where patients were asked to rate their pain, patients reported that drugs relieved their pain only 9% greater than placebo.^17^ The placebo effect was greater than expected in this trial and women in the control group may have rated their pain lower in response to an expected treatment response.

The more promising finding in this study was a significant reduction in breast nodularity in the experimental group, in particular, a 53% decrease in the number of women with the most severe breast nodularity (Table 4) where breast pain is shown to be the greatest. In addition, the significant correlation between nodularity and breast pain indicated that greater number of nodules was associated with higher breast pain (Table 5). Although the reduction in nodularity may have been anticipated from prior studies of iodine in preclinical models and in women with FBC, these studies were conducted using different forms of iodine at doses substantially higher than contained in this study formula.^7,8^ Reduced nodularity was also noted in an ormeloxifene trial.^18^

In the trials of active hormonal agents such as danazol^13^ and goserelin,^5^ breast nodularity was reduced before relief of pain. It may be that the current trial is showing similar early benefits of nodularity reduction. The clinical relevance of nodularity reduction may facilitate physical breast examination for fibrocystic changes and improve the screening value of mammograms in women with severely nodular breasts. This is in line with the well-known observation that the sensitivity of screening mammography increases with age, due to the reduction in breast density with increasing age. It is interesting to note that the reduction in overall breast nodularity in the experimental group corroborated with the previous demonstration of synergistic effects of GLA, iodine, and selenium on strengthening intracellular TJ function for preventing swelling and edema, the etiology of breast nodularity.^11,12^

Both groups of women reported a reduced need for nonprescription medications for control of their breast pain; however, in those women who continued pain medication, the women in the experimental group used significantly less pain medication by the end of the study (Fig. 3). The greater use of pain medication by women in the control group suggests that the reduction of breast pain attributed solely to the control nutritional formulation may have been overestimated. This difference was further demonstrated when the reduction in breast pain was reported as a function of the amount of pain medication taken (or APS). It is reasonable to assume that a reduction in analgesic requirements is a surrogate for a reduction in pain.

In further evaluation of overall impression of change in pain (PGIC), women in both groups rated their breast pain similarly by the end of the study. Approximately three-quarters of the women rated their pain as “improved” and half described their condition as “much or very much improved.”

The amount of iodine in the experimental nutritional formulation was based on the tolerable upper limit (TUL) for iodine intake of 1100 μg per day.^19^ The median intake of iodine from food for women in the United States is estimated as 190–200 μg per day, with the 90th percentile at 460 μg per day (NHANES III).^20^ Based on these estimates and with the experimental product containing 750 μg of iodine per daily serving (4 fl oz), the total amount of iodine from the diet and study formula was below the TUL in majority of women in the general population. The amount of iodine in the experimental formula was also significantly less than that in other forms of iodine used in the treatment of cyclic mastalgia.^7^

In general, the experimental formulation was well tolerated with no serious adverse events compared to control. Moreover, there was no significant difference in weight changes between the groups at the end of the trial. Since the experimental formulation contained approximately fourfold more iodine relative to the typical daily dietary intake, elevated TSH levels in four women attributable to the study product was largely expected. The mild transient elevation of TSH levels would be anticipated by the Wolff-Chaikoff effect that allows the human thyroid to adapt to dynamic changes in iodine intake.^21^ For most women, this change in TSH would be expected to resolve with continued intake of iodine, as was observed in the study.

The limitations of the study are that women in both groups had relatively low pain scores at baseline (Table 2), making it less likely that pain scores would be substantially further reduced and more difficult to demonstrate a separation in pain reduction between control and treatment groups; the long duration of FBC symptoms in women before study entry (means of 8.5 and 9.5 years) may have contributed to the development of a greater level of pain tolerance resulting in low levels of pain scores at baseline, which allowed women to accommodate to the discomfort or develop pain tolerance; and failure of the chosen methodology to effectively measure the symptom described as pain, despite well-documented reduction in pain medication by IVRS. Reconsideration of the appropriate control formulation for this type of study may also be required. The choice of HOSO enriched with oleic acid in the isocaloric, isonitrogenous control formulation may have contributed to the reduction in breast pain in women in the control group. Recently, oleic acid has been shown to inhibit pain response in mouse models of sepsis^22,23^ and reduce proinflammatory cytokines associated with pain in humans.^24^

Strengths of the study are the intervention, which for the first time combined the two ingredients, GLA and iodine, known to reduce mastalgia; the reduction in pain medication often associated with gastrointestinal symptoms; and the observed reduction in pain medication, which is more likely to be correct than in other studies where assessment was less rigorous than IVRS, such as recall or tablet counts at the end of the month.

Recommendations for future studies with similar endpoints include enrolling women with breast pain scores of 6.0 or greater with a duration of continuous pain of no more than 1 to 2 years. A generic Likert scale for pain may not be sensitive enough for cyclic breast pain analysis. In women with FBC, the variable nature of breast pain combined with a lack of specific standardized tools for its measurement complicates clinical trial design and outcomes. Thought should be given to the development of a pain scale specific for cyclic breast pain. Reconsideration of the appropriate control formulation without high-oleic-safflower oil or standard of care should be considered for the control group. Including subjects taking oral contraceptive pills could confound the pain results. Women on oral contraception (OC) achieved nonsignificant greater reductions in pain versus women on other forms of contraception, or who were not sexually active (Table 3). Moreover, women on OC also appeared to have numerically higher pain scores at baseline. Future studies could a priori plan to balance randomization with regard to OC and/or analyze the group taking oral contraceptive pills separately. This study was initiated before the publication of the Lucknow-Cardiff breast nodularity scale, which should be used in all future studies determining breast nodularity. Some women attending clinic seeking reassurance for absence of cancer may benefit from a cooling down period of 2 months. Inclusion of only those women whose pain perseveres with severity after 2 months may be beneficial. Consider enrolling women willing to discontinue the use of OTC pain medication during the trial.

In conclusion, women with FBC who consumed the nutritional formula with GLA, iodine, and selenium showed significantly less breast nodularity, a condition associated with less cyclic breast pain. In women who continued to use nonprescription medications for breast pain, a decrease in the quantity of nonprescription pain medication was observed. A nonpharmacological, noninvasive, well-tolerated, nutritional approach with anti-inflammatory and antipain activity and few side effects is likely to be appealing to women experiencing mastalgia. A larger study that is focused on the ability of the experimental formula to reduce nodularity and mammographic density as primary outcomes in women with FBC is warranted.
